# Proximate Composition and Nutritional Attributes of Ready-to-Cook Catfish Products

**DOI:** 10.3390/foods10112716

**Published:** 2021-11-06

**Authors:** John M. Bland, Casey C. Grimm, Peter J. Bechtel, Uttam Deb, Madan M. Dey

**Affiliations:** 1 USDA, Agricultural Research Service, SRRC, New Orleans, LA 70124, USA; casey.grimm@usda.gov (C.C.G.); peter.bechtel@usda.gov (P.J.B.); 2 Department of Aquaculture and Fisheries, University of Arkansas at Pine Bluff, Pine Bluff, AR 71601, USA; debu@uapb.edu; 3 Department of Agricultural Sciences, Texas State University, San Marcos, TX 78666, USA; mmd120@txstate.edu

**Keywords:** catfish products, strips, fillet, Delacata, proximate composition, minerals, amino acids, fatty acids

## Abstract

To increase the demand for U.S. farm-raised catfish, five healthy, convenient ready-to-cook products were developed to expand consumers’ options beyond basic fresh or frozen fillets. Five new catfish products were produced, consisting of one hundred samples of each, including three size-types of Panko-breaded fish products (strips, center cuts of regular fillets, and center cuts from Delacata fillets) and two marinated products (sriracha and sesame-ginger). The breaded products were to be prepared by baking for convenience over traditional frying methods, while the marinated products were to be microwaved as healthy and convenient products. The nutrient content of the samples was analyzed, including protein, moisture, fat, fiber, ash, and carbohydrate, as well as minerals, amino acid, and fatty acid constituent content, with associated atherogenic index (AI) and thrombogenic index (TI), showing unique differences between the Panko-breaded and marinated products. In addition, a trend was observed showing an increase in moisture, protein, ash, and carbohydrate percentages, and a decrease in lipid content related to the volume-to-surface-area ratio, having the order of strips < standard fillets < Delacata fillets.

## 1. Introduction

The healthy eating trend has promoted fish as an important source of protein, essential nutrients, and trace elements with low fat content [1]. There has been over a 122% worldwide increase in total fish consumption or a 54% increase per capita from 1990 to 2018 [2]. In the United States, per capita fish consumption from 1990 to 2018 has increased by less than 0.1% [3].

Catfish is the largest segment of U.S. aquaculture [4], but imports of tilapia and pangasius have reduced the share of U.S. catfish consumed from 80% in 2002 to 24% in 2019 [5,6]. In 2008, the National Fisheries Institute reported [7] that U.S. per capita consumption of tilapia and pangasius was 1.2 and 0 lb, respectively, while consumption of U.S. Catfish was 0.92 lb. By 2012, U.S. catfish consumption had decreased to 0.5 lb per capita, while imported tilapia and pangasius had grown to 1.48 and 0.63 lb per capita, respectively. In 2019, U.S. per capita consumption of tilapia and pangasius had decreased to 0.98 and 0.36 lb per capita, respectively, while U.S. Catfish had increased slightly to 0.52 lb per capita [8].

The most important motivation for eating fish is taste and health, with bones and price being deterrents [9]. While crispy fried catfish, with cornmeal breading, is the traditional way catfish is eaten, the health-conscious consumer looks for alternative catfish products prepared by cooking methods that do not include the added calories or nutritional loss caused by deep-frying. It is also known that high-temperature cooking may cause certain types of cancer [10] and the production of acrylamide [11]. Alternative to frying, other methods of cooking would be baking, grilling, broiling, microwaving [12], or cooking in a pouch [13]. Consumption of baked or broiled fish has been shown to positively influence brain health [14]. However, battered/breaded fish products made for baking are also known to have a texture that is not as crisp as fried fish. Par frying can be used to improve texture properties of baked foods, but this would increase equipment and operation expenses, and, as with deep-frying, results in higher calories from the adsorbed oil in the product [15]. A baked product that is not par fried, but has the texture properties of a fried product, has the potential to meet consumer desire for convenient products and the purchasing specifications for food service operations, such as school districts, health care facilities, and government purchases.

Marination is also a simple method to improve the flavor, tenderness, and juiciness of meats. Marination can be used in several cooking methods, such as microwave, grilling, and roasting. In addition, the chemical composition of marinated meat will have increased polyphenolic antioxidant concentrations that have been shown to reduce heterocyclic amine formation, providing reduced exposure to carcinogens formed during high-temperature cooking [16].

To expand the demand for catfish, the design of products should match consumer preferences. The development of catfish products with a higher convenience level can increase demand for U.S. farm-raised catfish and better compete with other products—tilapia, salmon, tuna, etc. [17]. Examination of the websites of eleven U.S. farm-raised catfish certified processors shows that the products available are usually fresh or frozen raw products of different cuts, and sometimes a cornmeal-breaded product, made for frying, or a marinated product, either Cajun or lemon-pepper seasoned.

In this study, we report the development of five ready-to-cook catfish products for use as a baked and microwaved meal and determine their chemical/nutrient composition, including proximate, minerals, amino acid, fatty acid, and the related health lipid indices (AI, aetherogenic and TI, thrombogenic), with comparison between products. Product attributes related to sensory and health benefits are dependent on chemical and nutrient composition. The authors have previously reported [18,19] the sensory evaluation of these products, where a total of 121 catfish consumers evaluated the products’ visual (appearance, color, glossiness and serving size) and other organoleptic (smell, taste, in-mouth texture, smell intensity, saltiness, oiliness, crispiness and juiciness) properties and expressed their willingness to pay for the products. All products were found to be acceptable, with the baked Panko products preferred, especially the Delacata fillet cut.

## 2. Materials and Methods

Individually quick frozen (IQF) catfish fillets (containing phosphate) were purchased from a commercial catfish processor in Mississippi. Two types of fillet were used—a standard 7–9 oz (200–250 g) fillet and a 5 oz (140 g) Delacata fillet (prepared from larger fillets with deep skinning). Products to prepare the Panko breading and marinades were purchased from Walmart and included (A) Frontier Ground Ginger Root Powder, (B) McCormick Onion Powder, (C) McCormick Culinary Garlic Powder, (D) Kikkoman Less Sodium Soy Sauce, (E) Lee Kum Kee Pure Sesame oil, (F) McCormick Honey Sriracha Seasoning, (G) Heinz apple cider vinegar, (H) Progresso Panko Plain Crispy Bread Crumbs (wheat), (I) Zatarain’s New Orleans Style Creole Seasoning, and (J) Bertolli extra light Olive Oil.

Fillet and marinade preparation. The following products were prepared:

- 110 g standard catfish fillet pieces with a panko breading to be baked before serving—one piece per package.
- 110 g Delacata catfish fillet pieces with panko breading to be baked before serving—one piece per package.
- 40 g pieces of standard catfish fillet with panko breading to be baked before serving—two pieces per package.
- 110 g Delacata catfish fillet with approximately 2 oz of SRRC developed Sriracha marinade to be microwaved—one piece per package.
- 110 g Delacata catfish fillet with approximately 2 oz of SRRC developed Ginger marinade to be microwaved—one piece per package.

Fillets were partially thawed by tempering in a refrigerator (34–38 °F) overnight. The standard 7–9 oz fillets (approx. 232 g) were cut to produce a rectangular piece (approx. 110 g) from the center of the fillet. The 5 oz Delacata fillets (approx. 160 g) were cut to produce a rectangular piece (approx. 110 g) from the center of the fillet. Two fillet strips (approx. 40 g each) were cut from the first muscle ridge on either side of the centerline (head to tail) of a standard fillet. Delacata fillets are considered a premium, thick fillet product that has undergone deep skinning, resulting in less skin and underlying fat layer, giving a more uniform color and superior appearance.

Two marinades were prepared, (1) a ginger marinade [17.5 tsp (5.8 tbsp, 86 mL) ginger powder (A), 17.5 tsp (5.8 tbsp, 86 mL) garlic powder (C), 52.5 tbsp (776 mL) onion powder (B), 23.3 cup (5592 mL) soy sauce (D), and 35 tbsp (518 mL) sesame oil (E)] and (2) a spicy sriracha marinade [525 g honey sriracha (F), 4200 mL soy sauce (D), 525 mL sesame oil (E), 210 mL vinegar (G), and 840 mL water].

Three Panko products were prepared using standard fillets, Delacata fillets, or fillet strips. They were immersed in extra light olive oil (J) followed by rolling the samples in a mixture of Panko wheat bread crumb (H) and a Cajun spice blend (I) (5:1) to enhance the flavor. Individual samples were placed in small plastic bags that were heat sealed and frozen.

For the marinated products, Delacata fillet cuts were placed in small plastic bags with 2 fl. oz of the marinade, heat sealed, and allowed to marinate in a refrigerator for up to 120 min before freezing.

Chemical Analyses. Analyses on raw products were conducted by the University of Missouri-Columbia, Agricultural Experiment Station Chemical Laboratory. Proximate Analyses included crude protein (CP), determined by combustion analysis (736 Nitrogen, LECO, St. Joseph, MI, USA) using AOAC Official Method 990.03, 2006. CP was reported from the calculation: 6.25× Nitrogen value. Ash was determined using a Thermolyne 20,400 programmable furnace (Barnstead Thermolyne, Dubuque, IA, USA) by AOAC Official Method 942.05, 2006. Crude fat (CF) was determined by hexane Soxhlet extraction. Moisture (M) was determined gravimetrically with a vacuum oven by AOAC Official Method 934.01, 2006. Total carbohydrates were determined ‘by difference’ using the calculation: 100%-%(CP + Ash + CF + M). Amino acid analysis was conducted according to the AAOC Official Method 982.30 E(a,b,c), chp. 45.3.05, 2006, with tryptophan by alkaline hydrolysis according to AOAC Official Method 988.15, chp. 45.4.04, 2006, employing cation-exchange chromatography (Beckman 6300 Amino Acid Analyzer, Beckman Instruments, Fullerton, CA, USA) coupled with post-column ninhydrin derivatization and quantitation. Fatty Acid Profile-saturated, mono- and poly-unsaturates, was conducted by converting crude fat to fatty acid methyl esters with boron trifluoride using AOAC Official Method 969.33. Analysis by capillary GC (Varian 3400CX, Varian Associates, Park Ridge, IL, USA) equipped with dual flame ionization detectors was conducted by AOAC Official Method 996.06. Mineral analysis included calcium, magnesium, zinc, copper, iron, manganese, sodium and phosphorus by gravimetric AOAC Official Method 966.01 using an Applied Research Laboratories sequential ICP spectrometer equipped with a minitorch (Fisons, Beverly, MA, USA).

The atherogenic index (AI) and the thrombogenic index (TI) were calculated using fatty acid data by the following equations according to Ulbricht and Southgate [20]:

$$\mathrm{AI}=\frac{4\times C14:0+C16:0}{\mathrm{MUFA}+n6+n3}$$

$$\mathrm{TI}=\frac{C14:0+C16:0+C18:0}{0.5\times \mathrm{MUFA}+0.5\times n6+3\times n3+n3/n6}$$

where MUFA is the sum of all monounsaturated fatty acids, n6 is the sum of all polyunsaturated omega-6 fatty acids, and n3 is the sum of all polyunsaturated omega-3 fatty acids.

Cooking Methods. To bake the panko products, frozen samples were removed from the packaging and placed on baking sheets. Sheets with 2 fillets were placed in a convection oven (preheated to 300 °F) for approximately 30 min to obtain an internal temperature of approx. 165 °F (determined with temperature probes). After removing the sheets from the oven, and a final sit of approx. 3–4 min, an endpoint temperature of 171 °F was reached.

For microwave cooking of marinated products, the frozen bag was perforated in three places and placed on a dish in a microwave and cooked at a 1000 W setting for 5–6 min. The outside temperature of the fillet pieces was immediately determined with an infrared thermometer, with a desirable endpoint temperature of 165 °F.

Statistical analysis. Data were analyzed by one-way analysis of variance (ANOVA) with the Holm–Sidak post-test for comparison of differences among the means of samples, performed using the SigmaPlot statistical analysis program (Version 13.0, SPSS Inc., Chicago, IL, USA). A significance level of 0.05 was employed. Statistical results were shown in tables as the mean value ± SD.

## 3. Results and Discussion

Oil and panko breading were determined to add an average of 15% to the weight of the fillets and 19% to the weight of the strips. Based on proximate data, the calculated product weight increase was from 7.2% panko and 7.8% oil, and the strips weight increase was from 7.5% panko and 11.5% oil. Marinades added an average of 16% to the weight of the fillets. Higher-quality foods are normally related to increased nutritional and compositional factors. Physical structure, taste, and nutrient availability are dependent on chemical composition. Sensory differences between the described products should be based on differences in the composition or nutrients of either the underlying catfish or the oils/breading/spices added to the catfish.

### 3.1. Proximate Analysis

Proximate analysis (Table 1) showed that moisture content was highest in the marinated products, but a trend was also seen related to the volume-to-surface-area ratio of the Panko samples (strips < fillets < Delacata). The Sriracha sample was higher than the Ginger sample, possibly due to the additional water and vinegar in the Sriracha marinade. Reported values for untreated catfish samples [21,22] are approximately 78–81% moisture, slightly higher than the marinated products with some added carbohydrate, but significantly higher than the Panko products that had added fat and carbohydrate.

The protein content varied slightly between the five products, with Panko Delacata having the highest amount (13.8%), and the Siracha marinade being lowest (12.4%). Since all products except the Panko strips had the same volume of fish, the absolute protein content of these should be similar, with differences attributed to the differing additional components (increased moisture in the marinates or increased carbohydrate and lipid in the Panko products). The three Panko products were not significantly different from each other, and the two marinates were not significantly different, while there was an overlapping difference in percent protein between Panko and marinate products. Raw catfish fillet has been reported [21,22] to have approximately 15–17% protein, but normally will vary according the actual amount of fat present. With added fat and carbohydrate from the treatments, the percent protein should be decreased from the raw fillet.

There was a significant difference between the lipid content of the Panko-breaded products (5–10%) and the marinated products (2–3%). Additionally, a significant difference was seen within the Panko-breaded products related to the surface-area-to-volume ratio, with the strips having the highest fat content (9.8%) and Delacata with the lowest (5.3%). The differences in percentage of lipid was primarily offset by moisture content. Reported values for raw fillets [21,22] are approximately 4–6%. Fat content can be variable, depending on the length of depuration before harvest, where lack of feeding can cause a dramatic loss of fat content. The Panko Delacata fillet was within this range, even after addition of fat in the treatment. The two marinated products were lower than the range, possibly due to the increased amount of carbohydrate and ash content. The Panko strips were much higher than the range due to the large ratio of fat added in respect to the volume of the strip.

Ash content was not significantly different for all three Panko beaded products (~2.7%) while the ash was higher for both marinated products (~4.1%). Raw fillet is reported [21] to have approximately 1% ash, showing the amount added with the treatments. Conversely, carbohydrate was higher for the three Panko-breaded products (~7.5%) and lower for the marinated products (3.3% and 4.3%). Although not significant, the general trend within Panko products was similar to the percent lipid. Fiber was also determined, and all products contained a relatively low amount of fiber (<0.08%) except the Ginger with 0.11% fiber.

Since the Delacata cut theoretically has more fat removed during the skinning process, this should make the percent protein larger, but the Panko products have olive oil added that will increase the lipid content and decrease protein and other percentages. Even though not significantly different, the protein content of five products was unusual. The Panko strips had a larger percent protein than the fillets even though the ratio of volume of meat to breading is less. For the three Delacata products, Panko, Sriracha, and Ginger, the protein content was higher in the Panko product even though it also had higher amounts of lipid and carbohydrates from the breading. However, this is probably a result of the differences in moisture. If all percentages are converted to a dry-weight basis, as in Table 2, the protein values are more easily compared. The percent protein is now significantly different between the Panko products and is smaller for the strips, compared to the fillets, in relation to the decreased meat volume. There is a similar percentage of protein in the Ginger and Sriracha marinated products that is significantly larger than the Panko Delacata, resulting from its added breading components. Lipid shows a similar trend to that found in Table 1, but ash and carbohydrate show a trend increasing from strips to fillets and Delacata.

### 3.2. Mineral Analysis

Sodium was the most abundant mineral in the samples, with Sriracha having the highest amount of 6% and the Panko strips and fillet having the lowest with 2.5% (Table 3). It should be noted that the IQF fillets that all products were prepared from, contained sodium polyphosphates as an additive. Mineral content, except for copper, was greater in the marinated products than in the Panko-breaded products, ranging, on average, from 1.3 times for phosphorus to 2.7 times for calcium (Mg = 2.2×, P = 1.3×, K = 1.5×, Na = 2.2×, Ca = 2.7×, Cu = 0.8×, Fe = 1.9×, Zn = 1.6×). Between the two marinated products, Ginger had significantly more of most mineral elements. Compared to reported values [23,24] of raw catfish fillet mineral content (0.10%, 0.71%, 1.38%, 0.16%, 484 ppm, 1.19 ppm, 42.1 ppm, and 27.3 ppm for Mg, P, K, Na, Ca, Cu, Fe and Zn, respectively), sodium was higher in the panko products and calcium, iron, and zinc were lower. In the marinates, sodium was higher and iron was lower. A relatively low content of copper (0.66 ppm) was seen in the sriracha product. The products represent a good source of minerals including potassium, zinc, and others.

### 3.3. Amino Acid Profiles

As seen in Table 4, all proteinogenic amino acid content had a general increasing trend for the Panko products related to strips < fillets < Delacata that correlated with the volume-to-surface-area ratio trend increase for these products. Additionally, there was a further increase in the content of each amino acid for the marinated products, except for tryptophan and tyrosine. The major amino acid was glutamic acid, followed by aspartic acid, lysine, leucine, and arginine. There have been various reports that glutamic and aspartic acid were major amino acids in other fish species such as Pacific Ocean perch [25] and yelloweye rockfish [26]. The hydrophobic amino acids alanine, valine, isoleucine, glycine, phenylalanine, and proline, as well as serine and threonine were of similar lower content. Histidine, methionine, and tyrosine were at similar lower levels, followed by cysteine, tryptophan, taurine, and hydroxyproline. The essential amino acid content was approximately 41% of the total amino acid content, for all samples. Lysine and leucine were the most abundant (3–5%). In comparison to the reported amino acid content of raw catfish fillets [23], all products showed a larger amount of glutamic acid and isoleucine, with a much lower percent amount of methionine. In addition, the Panko products showed a much smaller amount of serine and threonine, and a lower decrease in arginine and histidine. The higher content of proline, glycine, and hydroxyproline in the marinated samples are consistent with a greater content of collagen (an animal protein). Overall, the amino acid profile indicated the products are a good source of high-quality protein.

### 3.4. Fatty Acid Profiles

The fatty acid profile (Table 5) of the five products showed oleic acid to be the predominant fatty acid, with 90% of the total fatty acid content consisting of palmitic, stearic, oleic, and linoleic acids. The Atlantic salmon and [27], and Pacific Ocean perch [25] showed similar abundant fatty acids. In the Panko products, the oleic acid concentration for the strips and Delacata were not significantly different, at 61% and 62%, while the fillet was significantly different from the other two, at 56%. A similar difference was seen for next two abundant fatty acids, palmitic acid and linoleic acid, except instead of having a lower value, the fillet value was higher than the other two. The oleic acid concentrations for the Panko products were much higher than the reported [23,24] value of catfish fillets of approximately 43%. These high concentrations for oleic acid and relatively low linoleic acid concentration for all three Panko products was similar to that of the olive oil used for the Panko breading, which has oleic acid and linoleic acid concentrations of 78% and 7%, respectively. The values for oleic acid and linoleic acid for the Panko Delacata were 62% and 11%, respectively.

The fatty acid profile of the marinates showed oleic acid and linoleic acid concentrations of 38% and 28%, respectively. The oleic acid was reduced, and the linoleic acid was increased, with respect to the Panko products and to the raw fillet [23,24]. Palmitic acid was also increased slightly to 15%. These percentages were more similar to that of soy (23% and 51% for oleic and linoleic acids, respectively) and sesame (45% and 40% for oleic and linoleic acids, respectively) oils used in the marinades. Omega-3 fatty acids were at low concentrations of approximately 1–2% for the five products, while omega-6 concentrations were approximately 12–13% for the Panko products and 30% for the marinated products. The omega-3/omega-6 ratio was approximately 0.1 for all products. However, the products are shown to be a good source of polyunsaturated fatty acids. Polyunsaturated fatty acid percentages of the five products were higher than many meats, a characteristic of healthier meat, having health benefits such as decreasing stroke risk, triacylglycerol levels, blood pressure, insulin resistance, and inflammation, and might be useful as part of therapy for COVID-19 [28]. The atherogenic index (AI) shows slight differences between products, with values being slightly less than for gilthead sea bream (~0.25) [29], but lower than carp or rainbow trout with values of approximately 0.52 and 0.63, respectively [30]. The thrombogenic index (TI) values had more significant differences between products but not correlated to treatment or size. Values were higher than found for gilthead sea bream (~0.18) [29], but lower than carp (0.63) [30], and similar to rainbow trout (0.49) [30]. A healthy diet is characterized by low AI and TI values. Low AI and TI are good for retarding atherosclerosis and thus risk of cardiovascular disorders [31], whereas low TI decreases the threat of atrial fibrillation and the risk of stroke [32]. Harvard’s Healthy Eating Pyramid [33] recommends a polyunsaturated to saturated fat ratio of ≥ 1, for which the marinates have met. The presence of certain fatty acids has also been found to correlate with texture characteristics [34].

## 4. Conclusions

This study introduced five newly developed convenient catfish products. The compositional and nutrient characteristics were determined, showing differences between the Panko-breaded and marinated products. In addition, a general trend related to the size/thickness of the product was observed, where increasing the volume-to-surface-area ratio (strips < standard fillets < Delacata fillets) correlated to an increase in moisture, protein, ash, and carbohydrate percentage, and a decrease in the lipid percentage. Minerals, amino acid, and fatty acids showed similar differences between Panko and marinated products and showed several healthy attributes.

## Figures and Tables

**Table 1:** Proximate compositions (wet weight basis).

	Panko Strips	Panko Fillets	Panko Delacata	Sriracha	Ginger
Moisture	66.1 (±1.5) ^a^	68.9 (±1.5) ^b^	70.8 (±1.1) ^c^	77.4 (±0.7) ^e^	76.2 (±0.8) ^d^
Protein †	13.8 (±0.5) ^b^	13.4 (±0.6) ^b^	13.9 (±0.6) ^b^	12.4 (±0.5) ^a^	13.0 (±0.5) ^ab^
Lipid	9.8 (±1.5) ^d^	7.4 (±1.4) ^c^	5.3 (±0.6) ^b^	2.7 (±0.3) ^a^	2.4 (±0.7) ^a^
Ash	2.8 (±0.2) ^a^	2.7 (±0.2) ^a^	2.7 (±0.1) ^a^	4.2 (±0.3) ^b^	4.0 (±0.1) ^b^
Carbohydrate *	7.7 (±0.7) ^c^	7.6 (±0.3) ^c^	7.2 (±0.5) ^c^	3.3 (±0.3) ^a^	4.3 (±0.2) ^b^

Values are average (*n* = 8) percentages on a wet weight basis, with standard deviation in parentheses. † Protein = %N × 6.25. * Carbohydrate is calculated as the difference from 100% total for each sample replicate. Sriracha and Ginger are Delacata fillets. Values with different letters in a row are significantly different (*p* < 0.05).

**Table 2:** Proximate compositions (dry weight basis).

	Panko Strips	Panko Fillets	Panko Delacata	Sriracha	Ginger
Protein †	40 (±3) ^a^	43 (±3) ^b^	47.7 (±1.5) ^c^	54 (±3) ^d^	54.7 (±1.2) ^d^
Lipid	29 (±3) ^e^	24 (±3) ^d^	18.0 (±1.6) ^c^	13 (±3) ^b^	10 (±2) ^a^
Ash	8.4 (±0.7) ^a^	8.7 (±0.6) ^ab^	9.4 (±0.6) ^b^	18.4 (±1.3) ^d^	16.8 (±0.8) ^c^
Carbohydrate *	22.8 (±1.6) ^c^	24.5 (±1.3) ^d^	24.9 (±1.2) ^d^	14.3 (±1.5) ^a^	18.6 (±1.1) ^b^

Values are average (*n* = 8) percentages on a dry weight basis, with standard deviation in parentheses. † Protein = %N × 6.25. * Carbohydrate is calculated as the difference from 100% total for each sample replicate. Sriracha and Ginger are Delacata fillets. Values with different letters in a row are significantly different (*p* < 0.05).

**Table 3:** Mineral compositions.

	Panko Strips	Panko Fillets	Panko Delacata	Sriracha	Ginger
Magnesium (%)	0.064 (±0.001) ^a^	0.068(±0.001) ^b^	0.075 (±0.001) ^c^	0.144 (±0.002) ^d^	0.164 (±0) ^e^
Phosphorus (%)	0.640 (±0.001) ^a^	0.768 (±0.02) ^b^	0.803 (±0.003) ^c^	0.928 (±0.007) ^d^	0.929 (±0) ^d^
Potassium (%)	0.845 (±0.006) ^a^	0.88 (±0.02) ^b^	0.986 (±0.007) ^c^	1.29 (±0.02) ^d^	1.41 (±0.03) ^e^
Sodium (%)	2.52 (±0.02) ^a^	2.48 (±0.04) ^a^	2.74 (±0.02) ^b^	6.01 (±0.03) ^d^	5.29 (±0.04) ^c^
Calcium (ppm)	248 (±56) ^a^	236 (±8) ^a^	255 (±2) ^a^	593 (±8) ^b^	749 (±2) ^c^
Copper (ppm)	0.95 (±0.09) ^b^	1.20 (±0.10) ^c^	0.97 (±0.02) ^b^	0.66 (±0.05) ^a^	1.03 (±0.06) ^bc^
Iron (ppm)	14.2 (±5.4) ^ab^	11.6 (±2.4) ^ab^	10.5 (±2.5) ^a^	19.6 (±0.7) ^b^	26.7 (±1.9) ^b^
Zinc (ppm)	13.0 (±0.2) ^a^	14.4 (±0.2) ^b^	14.1 (±0.1) ^b^	20.6 ±0.5) ^c^	24.8 (±0.2) ^d^

Sriracha and Ginger are Delacata fillets. Values are averages (*n* = 3) on a sample dry weight basis, with standard deviation in parentheses. Values with different letters in a row are significantly different (*p* < 0.05).

**Table 4:** Amino acid compositions.

	Panko Strips	Panko Fillets	Panko Delacata	Sriracha	Ginger
Alanine	2.15 (±0.11) ^a^	2.43 (±0.02) ^b^	2.60 (±0.10) ^c^	3.18 (±0.05) ^d^	3.15 (±0.03) ^d^
Arginine	2.32 (±0.12) ^a^	2.61 (±0.06) ^b^	2.78 (±0.11) ^c^	3.36 (±0.03) ^d^	3.51 (±0.02) ^e^
Aspartic Acid ^1^	3.80 (±0.23) ^a^	4.18 (±0.17) ^a^	4.52 (±0.17) ^c^	5.38 (±0.03) ^d^	5.33 (±0.05) ^d^
Cysteine	0.47 (±0.03) ^a^	0.52 (±0.02) ^a^	0.56 (±0.02) ^b^	0.62 (±0.01) ^c^	0.62 (±0.00) ^c^
Glutamic Acid ^1^	6.32 (±0.35) ^a^	6.99 (±0.25) ^b^	7.63 (±0.21) ^c^	8.59 (±0.08) ^d^	8.83 (±0.03) ^d^
Glycine	1.84 (±0.07) ^a^	2.14 (±0.02) ^ab^	2.19 (±0.14) ^b^	2.61 (±0.10) ^c^	2.52 (±0.07) ^c^
Histidine *	0.87 (±0.04) ^a^	0.98 (±0.05) ^b^	1.05 (±0.04) ^c^	1.24 (±0.02) ^d^	1.25 (±0.01) ^d^
Hydroxylysine	0.03 (±0.01)	0.04 (±0.01)	0.04 (±0.01)	0.04 (±0.00)	0.04 (±0.00)
Hydroxyproline	0.28 (±0.03)	0.29 (±0.08)	0.28 (±0.04)	0.36 (±0.06)	0.33 (±0.10)
Isoleucine *	1.87 (±0.10) ^a^	2.09 (±0.10) ^b^	2.26 (±0.07) ^c^	2.68 (±0.01) ^d^	2.65 (±0.02) ^d^
Leucine *	3.04 (±0.16) ^a^	3.39 (±0.18) ^b^	3.67 (±0.13) ^c^	4.24 (±0.01) ^d^	4.20 (±0.01) ^d^
Lysine *	3.52 (±0.21) ^a^	3.94 (±0.20) ^b^	4.27 (±0.17) ^c^	4.88 (±0.01) ^d^	4.81 (±0.01) ^d^
Methionine *	1.00 (±0.06) ^a^	1.12 (±0.05) ^b^	1.21 (±0.04) ^b^	1.22 (±0.01) ^b^	1.21 (±0.01) ^b^
Phenylalanine *	1.62 (±0.09) ^a^	1.81 (±0.08) ^b^	1.97 (±0.07) ^c^	2.27 (±0.01) ^d^	2.28 (±0.01) ^d^
Proline	1.58 (±0.09) ^a^	1.75 (±0.06) ^b^	1.87 (±0.09) ^b^	2.18 (±0.09) ^c^	2.15 (±0.02) ^c^
Serine	1.42 (±0.06) ^a^	1.54 (±0.03) ^b^	1.65 (±0.05) ^c^	2.05 (±0.02) ^d^	2.08 (±0.03) ^d^
Threonine	1.73 (±0.09) ^a^	1.89 (±0.08) ^b^	2.05 (±0.08) ^c^	2.45 (±0.01) ^d^	2.43 (±0.01) ^d^
Tryptophan *	0.51 (±0.01) ^a^	0.57 (±0.02) ^b^	0.58 (±0.02) ^b^	0.56 (±0.01) ^b^	0.52 (±0.02) ^ab^
Tyrosine *	1.29 (±0.08) ^a^	1.42 (±0.07) ^ab^	1.56 (±0.06) ^b^	1.55 (±0.01) ^b^	1.50 (±0.01) ^b^
Valine *	1.98 (±0.11) ^a^	2.22 (±0.11) ^b^	2.40 (±0.09) ^c^	2.86 (±0.01) ^d^	2.82 (±0.01) ^d^
Total EAA	15.70 (±0.33)	17.54 (±0.33)	18.96 (±0.26)	21.50 (±0.03)	21.26 (±0.04)
Total NEAA	21.94 (±0.47)	24.38 (±0.39)	26.16 (±0.36)	30.81 (±0.18)	30.99 (±0.14)
Lanthionine §	0.08 (±0.02) ^a^	0.08 (±0.02) ^a^	0.06 (±0.01) ^a^	0.14 (±0.02) ^b^	0.12 (±0.01) ^b^
Ornithine §	0.03 (±0.00) ^a^	0.03 (±0.00) ^a^	0.04 (±0.00) ^b^	0.09 (±0.00) ^c^	0.10 (±0.01) ^c^
Taurine §	0.47 (±0.00) ^b^	0.53 (±0.02) ^c^	0.52 (±0.02) ^c^	0.46 (±0.01) ^ab^	0.44 (±0.01) ^a^
Total NPAA	0.58 (±0.02)	0.65 (±0.03)	0.63 (±0.02)	0.70 (±0.02)	0.66 (±0.01)

Values are average (*n* = 3) percentages on a sample dry weight basis, with standard deviation in parentheses. ^1^ Aspartic acid includes Asparagine; Glutamic acid includes Glutamine. § Non-proteinogenic amino acids (NPPA). EAA = Essential Amino Acids, NEAA = Non-Essential Amino Acids. * Identifies the nine essential amino acids. Sriracha and Ginger are Delacata fillets. Values with different letters in a row are significantly different (*p* < 0.05).

**Table 5:** Fatty acid profiles.

	Panko Strips	Panko Fillets	Panko Delacata	Sriracha	Ginger
Myristic acid (14:0)	0.39 (±0) ^a^	0.5 (±0.06) ^b^	0.37 (±0.01) ^a^	0.62 (±0.02) ^c^	0.64 (±0.05) ^c^
Pentadecylic acid (15:0)	0.05 (±0.01) ^a^	0.05 (±0) ^a^	0.04 (±0) ^a^	0.06 (±0.01) ^a^	0.08 (±0.01) ^b^
Palmitic acid (16:0)	13.77 (±0.15) ^a^	15.55 (±0.7) ^b^	13.19 (±0.09) ^a^	14.88 (±0.25) ^b^	15.52 (±0.42) ^b^
t-Palmitoleic acid (9t-16:1n7)	0.28 (±0.01) ^a^	0.32 (±0.02) ^b^	0.26 (±0) ^a^	0.3 (±0.01) ^b^	0.35 (±0.01) ^c^
Palmitoleic acid (9c-16:1n7)	1.23 (±0.02) ^a^	1.44 (±0.08) ^b^	1.29 (±0.01) ^a^	1.44 (±0.03) ^b^	1.49 (±0.06) ^b^
Stearic acid (18:0)	4.33 (±0.02) ^b^	4.89 (±0.05) ^d^	3.92 (±0.04) ^a^	4.78 (±0.04) ^c^	4.95 (±0.05) ^d^
Elaidic acid (9t-18:1n9)	0.12 (±0) ^a^	0.18 (±0.01) ^d^	0.11 (±0.01) ^a^	0.15 (±0) ^b^	0.16 (±0.01) ^c^
Oleic acid (9c-18:1n9)	61.32 (±0.19) ^c^	56.21 (±0.47) ^b^	61.73 (±0.09) ^c^	38.79 (±0.18) ^a^	38.4 (±0.3) ^a^
Vaccenic acid (11c-18:1n7)	1.75 (±0.04) ^b^	1.78 (±0.03) ^b^	1.97 (±0.01) ^c^	1.42 (±0.01) ^a^	1.44 (±0.01) ^a^
Linoleic acid (18:2n6)	10.74 (±0.03) ^a^	12 (±0.08) ^b^	10.73 (±0.02) ^a^	28.85 (±0.02) ^d^	27.22 (±0.17) ^c^
Linolenic acid (18:3n3) †	0.81 (±0.01) ^b^	0.74 (±0.03) ^a^	0.91 (±0) ^c^	0.82 (±0.01) ^b^	0.76 (±0.01) ^a^
γ-Linolenic acid {18:3n6)	0.15 (±0) ^b^	0.21 (±0.01) ^e^	0.13 (±0) ^a^	0.17 (±0) ^c^	0.2 (±0.01) ^d^
Arachidic acid (20:0)	0.3 (±0.01) ^b^	0.27 (±0.01) ^a^	0.33 (±0.01) ^bc^	0.35 (±0.02) ^c^	0.33 (±0.01) ^c^
Gondoic acid (20:1n9)	0.92 (±0.01) ^b^	1.13 (±0.05) ^c^	0.76 (±0.02) ^a^	0.88 (±0.04) ^b^	0.93 (±0.04) ^b^
Eicosadienoic acid (20:2n6)	0.41 (±0.01) ^b^	0.51 (±0.02) ^c^	0.34 (±0.01) ^a^	0.51 (±0.02) ^c^	0.6 (±0.02) ^d^
Eicosatrienoic acid (20:3n3) †	0.03 (±0.01) ^ab^	0.04 (±0.01) ^ab^	0.03 (±0.01) ^a^	0.05 (±0) ^b^	0.05 (±0.01) ^b^
Dihomo-γ-linolenic acid (20:3n6)	0.45 (±0.01) ^a^	0.64 (±0.02) ^b^	0.5 (±0.03) ^a^	0.8 (±0.03) ^c^	0.87 (±0.03) ^d^
Arachidonic acid (20:4n6)	0.39 (±0.01) ^a^	0.53 (±0.02) ^b^	0.52 (±0.02) ^bc^	0.84 (±0.04) ^c^	0.95 (±0.02) ^d^
EPA (20:5n3) †	0.09 (±0) ^a^	0.09 (±0) ^a^	0.12 (±0) ^bc^	0.2 (±0.01) ^c^	0.23 (±0.02) ^d^
Behenoic acid (22:0)	0.27 (±0) ^a^	0.35 (±0.02) ^b^	0.28 (±0.01) ^a^	0.35 (±0.01) ^b^	0.37 (±0.02) ^b^
Erucic acid (22:1n9)	0.06 (±0)	0.06 (±0)	0.05 (±0.02)	0.07 (±0.01)	0.07 (±0.02)
Docosadienoic acid (22:2n6)	0.08 (±0.01) ^ab^	0.09 (±0.01) ^b^	0.08 (±0.01) ^a^	0.11 (±0.01) ^c^	0.13 (±0.01) ^c^
Adrenic acid (22:4n6)	0.05 (±0) ^a^	0.07 (±0.01) ^b^	0.05 (±0.01) ^a^	0.08 (±0.01) ^c^	0.09 (±0.01) ^c^
Clupanodonic acid (22:5n3) †	0.1 (±0) ^a^	0.12 (±0.03) ^a^	0.13 (±0.01) ^a^	0.21 (±0.01) ^b^	0.25 (±0.02) ^b^
DHA (22:6n3) †	0.31 (±0.01) ^a^	0.33 (±0.02) ^a^	0.46 (±0.02) ^bc^	0.75 (±0.05) ^c^	0.89 (±0.06) ^d^
Lignoceric acid (24:0)	0.05 (±0.01) ^a^	0.05 (±0) ^a^	0.07 (±0.01) ^bc^	0.08 (±0) ^b^	0.08 (±0.01) ^b^
Nervonic acid (24:1n9)	0.03 (±0.01) ^ab^	0.06 (±0.02) ^b^	0.05 (±0.01) ^ab^	0.05 (±0.01) ^ab^	0.03 (±0.01) ^a^
Saturated fatty acids	19.16 (±0.15) ^b^	21.7 (±0.7) ^c^	18.20 (±0.1) ^a^	21.1 (±0.3) ^c^	22.0 (±0.4) ^c^
Monounsaturated FA	65.7 (±0.2) ^c^	61.2 (±0.5) ^b^	66.23 (±0.09) ^d^	43.11 (±0.19) ^a^	42.9 (±0.3) ^a^
Polyunsaturated FA	13.62 (±0.03) ^a^	15.37 (±0.1) ^c^	14.00 (±0.04) ^b^	33.39 (±0.07) ^e^	32.2 (±0.2) ^d^
n3/n6	0.11 (±0.00) ^d^	0.09 (±0.01) ^c^	0.13 (±0.00) ^e^	0.06 (±0.00) ^a^	0.07 (±0.00) ^b^
Atherogenic index (AI)	0.19 (±0.00) ^a^	0.23 (±0.01) ^b^	0.18 (±0.00) ^a^	0.23 (±0.00) ^b^	0.24 (±0.01) ^b^
Thromogenic index (TI)	0.43 (±0.00) ^b^	0.50 (±0.02) ^d^	0.39 (±0.00) ^a^	0.47 (±0.01) ^c^	0.49 (±0.01) ^cd^

Values are average (*n* = 3) percentages on a dry weight basis (expressed as percent of total lipid), with standard deviation in parentheses. † Omega-3 fatty acids. Sriracha and Ginger are Delacata fillets. Values with different letters in a row are significantly different (*p* < 0.05).
